# Point prevalence surveys of antimicrobial use among eight neonatal intensive care units in India: 2016

**DOI:** 10.1016/j.ijid.2018.03.017

**Published:** 2018-06

**Authors:** Sumanth Gandra, Gerardo Alvarez-Uria, Srinivas Murki, Sanjeev K. Singh, Ravishankar Kanithi, Dasaratha R. Jinka, Ashok K. Chikkappa, Sreeram Subramanian, Anita Sharma, Dhanya Dharmapalan, Hemasree Kandraju, Anil Kumar Vasudevan, Onkaraiah Tunga, Akhila Akula, Yingfen Hsia, Mike Sharland, Ramanan Laxminarayan

**Affiliations:** a Center for Disease Dynamics, Economics & Policy, New Delhi, India; b Rural Development Trust Hospital, Bathalapalli, Andhra Pradesh, India; c Fernandez Hospital, Hyderabad, Telangana, India; d Amrita Institute of Medical Sciences, Kochi, Kerala, India; e Sowmya Children’s Hospital, Hyderabad, Telangana, India; f Rural Development Trust Hospital, Kalyanadurgam, Andhra Pradesh, India; g Paramitha Children’s Hospital, Hyderabad, Telangana, India; h Fortis Hospital, Mohali, Punjab, India; i Yewale Hospital, Mumbai, Maharashtra, India; j St. Georges University, London, United Kingdom; k Princeton University, NJ, USA

**Keywords:** Point prevalence survey, Antimicrobial use, Neonates, NICU, India

## Abstract

**Background:**

Information about antimicrobial use is scarce and poorly understood among neonatal intensive care units (NICUs) in India. In this study, we describe antimicrobial use in eight NICUs using four point prevalence surveys (PPSs).

**Methods:**

As part of the Global Antimicrobial Resistance, Prescribing, and Efficacy in Neonates and Children (GARPEC) study, one-day, cross-sectional, hospital-based PPSs were conducted four times between 1 February 2016 and 28 February 2017 in eight NICUs. Using a standardized web-based electronic data entry form, detailed data were collected for babies on antimicrobials.

**Results:**

A total of 403 babies were admitted to NICUs across all survey days, and 208 (51.6%) were prescribed one or more antimicrobials. Among 208 babies, 155 (74.5%) were prescribed antimicrobials for treatment of an active infection. Among 155 babies with an active infection, treatment was empiric in 109 (70.3%). Sepsis (108, 49.1%) was the most common reason for prescribing antimicrobials. Amikacin (17%) followed by meropenem (12%) were the two most commonly prescribed antimicrobials. For community-acquired sepsis, piperacillin-tazobactam (17.5%) was the most commonly prescribed drug. A combination of ampicillin and gentamicin was prescribed in only two babies (5%).

**Conclusions:**

The recommended first-line antimicrobial agents, ampicillin and gentamicin, were rarely prescribed in Indian NICUs for community acquired neonatal sepsis.

## Highlights

• Approximately half (51.6%) of NICU babies received at least one antimicrobial agent.
• Amikacin was the most commonly prescribed antimicrobial agent followed by meropenem.
• The recommended first-line antimicrobial agents, ampicillin and gentamicin were rarely prescribed for community acquired neonatal sepsis.

## Introduction

Each year, an estimated 56,624 neonates die because of sepsis resulting from bacteria resistant to first-line antimicrobial agents (Laxminarayan and Bhutta, 2016). In India, Gram-negative organisms are the predominant pathogens isolated from neonatal bloodstream infections (Dharmapalan et al., 2017). Antimicrobial resistance rates in neonatal blood stream infections in India between 2000 and 2015 showed extremely high resistance rates to first-line antimicrobial agents (Dharmapalan et al., 2017). The median ampicillin resistance rate for common Gram-negative pathogens *Klebsiella pneumoniae* and *Escherichia coli* was 95.9% and 92.9%, respectively. Similarly, the median gentamicin resistance rate was 75% and 55.6% for *K. pneumoniae* and *E. coli*, respectively (Dharmapalan et al., 2017).

Antimicrobial resistance is a natural phenomenon but is accelerated by the overuse and misuse of antimicrobials. Given the high prevalence of infections by bacteria resistant to first-line agents, there is a need for better understanding of the current antimicrobial prescribing practices among NICU babies in India. However, information about antimicrobial use is scarce and comes mainly from single center studies (Suryawanshi et al., 2015, Chatterjee et al., 2007). In this study, we describe antimicrobial use in eight NICUs in India using four point prevalence surveys (PPSs).

## Methods

As part of the Global Antimicrobial Resistance, Prescribing, and Efficacy in Neonates and Children (GARPEC) study, one-day, cross-sectional hospital based PPSs were conducted four times between 1 February 2016 and 28 February 2017 in eight NICUs (Gandra et al., 2017). Four PPSs were conducted to improve the accuracy of antimicrobial use measurement and to examine the antimicrobial use variation at different time points. The first PPS was conducted between 1 February and 31 March 2016; the second between 1 May and 30 June 2016; the third between 1 September and 31 October 2016; and the fourth between 1 December 2016 and 28 February 2017. The date of the survey was chosen as per the convenience of the site principal investigator within the specified months. All babies present in the NICU at 8:00 a.m. were included in the survey and detailed data were recorded only for patients with an active antimicrobial prescription.

Among eight participating hospitals, five hospitals enrolled into the study at the time of initiation of the study on 1 February 2016. Three additional hospitals were enrolled by 1 May 2016 and did not participate in the first round of PPS. Among the eight hospitals, two were rural general trust hospitals, three were stand-alone private children’s hospitals, two were private tertiary care hospitals and one was a private mother and child-care center with inborn neonatal services. One tertiary care hospital, two stand-alone pediatric hospitals and the mother and child care center have teaching services in NICUs.

We used a standardized web-based electronic data entry form on the Research Electronic Data Capture (REDCap)^®^ developed for the GARPEC project to store de-identified patient data. For patients receiving antimicrobials (antibiotics, antifungal and antivirals, antiparasital agents), the following data were collected: patient sex, age, weight, ventilation status, comorbid conditions, number of antimicrobials, antimicrobial name, dose per administration, dose units, number of doses each day, route of administration, reason for treatment, treatment indication (community versus hospital acquired) or prophylaxis, and whether treatment was empirical or targeted after receiving a microbiological report. We included all diagnoses for which antimicrobials were prescribed even when there was more than one diagnosis. Ethics approval was obtained from the respective institutional human research ethics committees of participating hospitals.

Categorical variables were compared with the Chi-squared test or Fisher’s exact test, as appropriate. To investigate hospital characteristics associated with a higher proportion of patients on antimicrobials, we a performed multi-level mixed-effects logistic regression model with random intercepts for each site, including hospital type and presence of onsite diagnostic microbiology laboratory as covariates. Statistical significance was defined as *p <* 0.05. All statistical analyses were performed using STATA 14.2 (StataCorp, College Station, Texas, USA).

## Results

At the eight participating hospitals, the overall bed occupancy for all four-survey days was 71.3% and ranged from 50% to 100% (Table 1). A total of 403 babies were hospitalized across all survey days, and 208 babies were prescribed one or more antimicrobials (51.6%; 95% confidence interval (CI) 46.7% to 56.5%) (Table 1). The percentage of babies on antimicrobials ranged from 18.2% to 70.7%. Of 208 babies on antimicrobials, only one antimicrobial was prescribed in 83 babies (39.9%), two antimicrobials were prescribed in 111 babies (53.4%) and three or more were prescribed in 14 babies (6.7%). There were not significant differences among the four PPSs at study sites (data not shown).

**Table 1:** Hospital characteristics, bed occupancy and proportion of patients on antimicrobials (with Wilson 95% confidence interval).

Hospital	Hospital Type	Onsite Microbiology Laboratory	Total Number of Beds	Bed Occupancy (%)	Total Number of Patients	Number of Patients on Antimicrobials	Patients on Antimicrobials % (95% CI)
Hospital A	Tertiary	Yes	88	71.6	63	42	63.7 (54.4-77.1)
Hospital B**	Pediatric	No	16	50	8	6	75.0 (40.9-92.9)
Hospital C	Pediatric	Yes	122	73	89	31	34.8 (25.7-45.2)
Hospital D*	Tertiary	Yes	34	64.7	22	4	18.2 (7.3-38.5)
Hospital E	Pediatric	No	106	70.8	75	42	56.0 (44.7-66.7)
Hospital F*	General & Rural	Yes	84	79.8	67	29	43.3 (32.1-55.2)
Hospital G*	General & Rural	No	41	100	41	29	70.7 (55.5-82.4)
Hospital H	Pediatric	No	74	51.4	38	25	65.8 (49.9-78.8)
All			565	71.3	403	208	51.6 (46.7-56.5)

* These hospitals did not participate in the first point prevalence survey.

** This hospital did not participate in the second and fourth prevalence survey.

Onsite diagnostic microbiology services were available in four hospitals (Table 1), and antimicrobial treatment guidelines had been formulated in all hospitals. In the multi-level logistic regression model, having an onsite diagnostic microbiology laboratory was associated with lower odds of being on antimicrobials (adjusted odds ratio (aOR) 0.3; 95% CI 0.14 to 0.71), while there were no significant differences between pediatric hospitals (reference) and rural hospitals (aOR 1.45; 95% CI 0.63 to 3.3) or tertiary hospitals (aOR 1.75; 95% CI 0.62 to 4.96).

Of the 208 babies on antimicrobials 50% (104) were admitted to stand-alone pediatric hospitals, followed by rural hospitals (27.9%; 58), and tertiary hospitals (22.1%; 46) (Table 2). Sixty-eight babies (32.7%) were female and 42.3% (88) required invasive or non-invasive ventilation support (Table 2). The majority of babies (77.4%) had one or more co-morbidities. Of the 208 babies on antimicrobials in NICU, 185 (88.9%) were neonates, and 23 (11%) were older than one month. Among neonates, the median gestational age was 34.5 weeks (interquartile range [IQR], 31 to 38), and median birth weight was 1737 grams (IQR, 1210 to 2710).

**Table 2:** Characteristics of patients admitted to neonatal intensive care units.

Variables	N (%)
**Hospital type**	
Stand alone Pediatric	104 (50)
General Rural	58 (27.9)
Tertiary care	46 (22.1)
Female	68 (32.7)

**Ventilation support**	
Invasive	43 (20.7)
Non-Invasive	45 (21.6)
No support	120 (57.7)

**Co-morbidities**	
None	47 (22.6)
One	119 (57.2)
Two or more	42 (20.2)

Diagnosis*	
Sepsis	108 (49.1)
Newborn Sepsis Prophylaxis for Newborn Risk Factors	33 (15.0)
Proven or probable Bacterial LRTI	28 (12.7)
Newborn Sepsis Prophylaxis for Maternal Risk Factors	21 (9.5)
Treatment: for Surgical disease	7 (3.2)
Others	23 (10.5)

Indications*	
Community acquired	81 (36.8)
Hospital acquired	60 (27.2)
Prophylaxis	65 (29.5)
Unknown	14 (6.5)

**Empiric vs. Targeted Therapy (n** **=** 155)**	
Empiric	109 (70.3)
Targeted	46 (29.7)

Abbreviation: LRTI, lower respiratory tract infection.

* Total can be more than 100% as one patient can have more than one diagnosis.

** Only includes antimicrobial prescriptions for active infections (prescriptions for prophylaxis were excluded).

In 208 babies with an antimicrobial prescription, 143 (68.7%) were prescribed for treatment of active infection, 53 (25.5%) for prophylaxis, and in 12 (5.8%) babies for both active infection and prophylaxis (Table 2). Among 155 babies with an active infection, the treatment was empiric in 109 (70.3%) and targeted in 46 (29.7%). Sepsis (108, 49.1%), newborn prophylaxis for newborn risk factors (33, 15%), and lower respiratory tract infections (LRTIs) (28, 12.7%), were the three most common reasons for prescribing antimicrobials (Table 2 and Supplementary Table 1).

The five most common classes of antimicrobials prescribed were aminoglycosides (26.4%), third generation cephalosporins (14.1%), carbapenems (11.8%), piperacillin and enzyme inhibitor combinations (11.5%) and aminopenicillins (11.5%) (Table 3). Antifungals (azoles and amphotericin B) accounted for 6.6% of the prescriptions. Fifteen antimicrobial agents accounted for 90% of antimicrobial use (Table 4). The five most common antimicrobials prescribed were amikacin (17%), meropenem (12%), piperacillin-tazobactam (11%), ampicillin (10%), and gentamicin (9%). Piperacillin-tazobactam (27.7%), cefotaxime (15.7%), amikacin (15.7%) and meropenem (7.2%) were the antimicrobials most commonly used as monotherapy (Table 4). Ampicillin with gentamicin (21.6%), amikacin with piperacillin-tazobactam (10.8%), amikacin with ciprofloxacin (5.4%) and meropenem with vancomycin (5.4%) were the most common antimicrobial combinations used.

**Table 3:** Classes of antimicrobials prescribed by hospitals.

	Overall	Hospital A	Hospital B	Hospital C	Hospital D	Hospital E	Hospital F	Hospital G	Hospital H
Aminoglycosides	26.5	19.5	26.8	11.1	19.3	37.3	35.2	25.6	40
Third gen. Cephalosporins	14.1	15.9	9.8	0	24.6	2.0	14.8	9.3	50
Carbapenems	11.8	19.5	14.6	22.2	7.0	7.8	7.4	11.6	0
Piperacillin/beta-lactamase inhibitor	11.5	2.4	29.3	11.1	28.1	2.0	7.4	9.3	0
Aminopenicillins	11.5	15.9	2.4	0	1.8	23.5	24.1	0	0
Fluoroquinolones	6.9	7.3	0	11.1	1.8	19.6	0	14.0	0
Azole antifungal	4.9	8.5	2.4	0	1.8	0	7.4	9.3	0
Glycopeptides	3.5	3.7	12.2	11.1	1.8	2.0	1.9	0	0
Polymyxins	2.3	1.2	2.4	22.2	0	2.0	0	7.0	0
Amphotericin B	1.7	0	0	11.1	3.5	2.0	0	2.3	10
Others*	5.2	6.2	0	0	10.5	2.0	1.9	11.6	0

* Others include antivirals, glycylcycline, lincosamides, macrolides, metronidazole, oxazolidinones, penicillins, sulfonamides and trimethoprim.

**Table 4:** Most prescribing antimicrobials ranked by overall drug utilization 90% (DU90%).

	Overall	Monotherapy	Part of double combination therapy	Part of triple combination therapy
Amikacin	17.3	15.7	19.4	9.5
Meropenem	11.8	7.2	10.8	26.2
Piperacillin-tazobactam	11.2	27.7	6.3	4.8
Ampicillin	10.1	1.2	14.0	7.1
Gentamicin	9.2	3.6	13.1	0
Cefotaxime	5.8	15.7	3.2	0
Fluconazole	4.9	2.4	4.1	14.3
Ciprofloxacin	3.8	4.8	4.1	0
Vancomycin	3.5	1.2	4.1	4.8
Ceftriaxone	2.6	4.8	2.3	0
Colistin	2.3	0	2.7	4.8
Levofloxacin	2.0	2.4	2.3	0
Cefoperazone Sulbactam	3.2	0	4.5	2.4
Amphotericin B/Amphotericin B liposomal	1.7	2.4	1.4	2.4
Cefoperazone	1.4	1.2	1.4	2.4

Among 108 babies diagnosed with sepsis (57 community-acquired and 51 hospital-acquired), the most common antimicrobials prescribed were piperacillin-tazobactam (17; 15.9%), meropenem (7; 7.4%), combination of amikacin and piperacillin-tazobactam (7; 6.5%), amikacin (6; 5.6%), and ceftriaxone/cefotaxime (6; 5.6%). On reviewing empirical antimicrobial therapy for 40 babies with community-acquired sepsis, the five most common antimicrobial regimens prescribed were, piperacillin-tazobactam (7; 17.5%), combination of amikacin and piperacillin-tazobactam(5; 12.5%), combination of amikacin and ciprofloxacin (5; 12.5%), amikacin alone (5; 12.5%), and combination of amikacin and cefoperazone-sulbactam (3; 7.5%) (Supplementary Table 2). Combination of ampicillin and gentamicin was prescribed in only two babies (5%). Similarly, on reviewing empirical antimicrobial therapy for 28 babies with hospital-acquired sepsis the most common antimicrobial regimens prescribed were piperacillin-tazobactam (5; 17.9%), ceftriaxone/cefotaxime (3; 10.7%), fluconazole (3; 10.7%), combination of amikacin and meropenem (2; 7.1%) and combination of amikacin and piperacillin-tazobactam (2; 7.1%) (Supplementary Table 3).

On reviewing empirical antimicrobial therapy for 20 babies with community-acquired LRTI, the two most common antimicrobial regimens prescribed were cefotaxime (4; 20%) and piperacillin with enzyme inhibitor (3; 15%) (Supplementary Table 4). The antimicrobial regimens prescribed for newborn prophylaxis due to newborn risk factors include combination of ampicillin and gentamicin (13; 39.4%), piperacillin with enzyme inhibitor (3; 9.1%) and amikacin (2; 6.1%) (Supplementary Table 5). Similarly, the antimicrobial regimens prescribed for newborn prophylaxis due to maternal risk factors included combination of ampicillin and gentamicin (8; 38.1%) and amikacin (5; 23.8%).

## Discussion

To our knowledge, this is the first multi-center study describing antimicrobial use among NICU babies in India. In this study, approximately half (51.6%) of NICU babies received at least one antimicrobial agent and sepsis was the most common indication for prescribing antimicrobials. Amikacin was the most commonly prescribed antimicrobial agent followed by meropenem. Piperacillin-tazobactam was the most frequently prescribed empirical antimicrobial agent for community-acquired sepsis.

In the global Antibiotic Resistance and Prescribing in European Children (ARPEC) PPS study conducted in 2012, involving 226 hospitals from 41 countries, the overall percentage of neonates in NICU on antimicrobials was 30.3%, which was much lower than the 51.6% in our study (Versporten et al., 2016). Similar to global ARPEC study, sepsis was the most common reason for prescribing antimicrobials and aminoglycosides were the most commonly prescribed class of antimicrobials. However, the most commonly prescribed aminoglycoside was gentamicin, while in our study it was amikacin. Similar findings were observed in a three-year observational study involving two NICUs in Central India, where amikacin was the most commonly prescribed antimicrobial and comprised 18% of antimicrobial prescriptions (Hauge et al., 2017). The preference of amikacin over gentamicin might have been motivated by the susceptibility profile of Gram-negative pathogens in India. A recent review of antimicrobial resistance among bloodstream infections in neonates and children showed that the median gentamicin resistance rates were 75% and 55.6% for *K. pneumoniae* and *E. coli* respectively whereas median amikacin resistance rates were 41% and 22.4%, respectively (Dharmapalan et al., 2017).

The World Health Organization (WHO) and the Indian National Center for Disease Control (NCDC) recommend the combination of ampicillin and gentamicin as first-line empiric therapy for community acquired neonatal sepsis or pneumonia (National Center for Disease Control, 2016, Organization WH, 2012). However, we found that this combination was rarely used in the hospitals that participated in our study. Only two out of 40 babies with community-acquired sepsis and none of the 20 babies with community-acquired pneumonia were prescribed ampicillin and gentamicin. Instead, piperacillin-tazobactam or amikacin alone or combination of piperacillin-tazobactam and amikacin were commonly prescribed for community-acquired sepsis. For community-acquired pneumonia, cefotaxime as single agent and piperacillin-tazobactam alone were commonly prescribed. Ampicillin and gentamicin combination was only frequently prescribed for newborn prophylaxis due to neonatal or maternal risk factors. These antimicrobial prescribing practices might have been influenced by factors such as local antimicrobial susceptibility profile, availability and utilization of diagnostic microbiology services, severity of illness, and implementation of antimicrobial treatment polices. With the reported median ampicillin resistance rate more than 90% and median gentamicin resistance rate of more than 55% for *K. pneumoniae* and *E. coli*, the use of second line broad-spectrum agents may seem appropriate. However, with the high level of empiric therapy observed (71%), there could be greater inappropriate therapy. This is supported by a recent study in a single NICU, where implementation of antimicrobial policy based on microbiology information for neonatal sepsis in one of the participating hospitals (Hospital F) resulted in increased use of first-line agents and reduced use of third generation cephalosporins without effecting patient outcomes (Jinka et al., 2017). This highlights the importance of facility-based treatment guidelines, which are developed, based on the local microbiology data.

In our study, we observed that meropenem was the second most common antimicrobial agent prescribed after amikacin. It was mainly prescribed as empiric therapy for hospital-acquired infections as single agent or in combination with other agents. The use of meropenem may be indicated due to high prevalence of third generation cephalosporin resistant and extended spectrum beta-lactamase (ESBL) producing *Klebsiella* species and *E. coli*. This is evident by review of antimicrobial resistance rates among bloodstream infections in neonates between 2000 and 2015 (Dharmapalan et al., 2017). The median ceftriaxone resistance for *Klebsiella* species and *E. coli* was 65% and 50% respectively. However, with increased meropenem selection pressure, carabapenem resistant *Klebsiella* species have emerged and are increasingly encountered in Indian NICUs (Jinka et al., 2017, Investigators of the Delhi Neonatal Infection Study (DeNIS) Collaboration, 2016). There is an urgent need for carbapenem sparing agents to treat infections caused by ESBL-producing organisms to decrease the carbapenem selection pressure. Although, several studies suggest that piperacillin-tazobactam could be effective for infections by ESBL Enterobacteriaceae (Yoon et al., 2017, Gudiol et al., 2017), some studies indicate better survival rates with carbapenems (Tamma et al., 2015). Novel beta-lactam/beta-lactamase inhibitor combinations such as ceftazidime-avibactam and ceftolozane-tazobactam were non-inferior to carbapenems in treating invasive infections due to ESBL producing organism in randomized clinical trials (Wagenlehner et al., 2016, Popejoy et al., 2016). However, safety and efficacy data in neonates are not yet available. Meanwhile, implementing effective infection control practices is paramount to prevent the spread of these organisms in NICUs.

One interesting finding we observed in the study was that the proportion of babies on antimicrobials was lower in hospitals with onsite diagnostic microbiology laboratory, in spite of having antimicrobial treatment guidelines in all eight NICUs. This probably can be explained by the fact the utilization of microbiological culture results helped in de-escalating or stopping the empiric antimicrobial therapy early, especially among patients with possible and probable sepsis, and thus reduce the number of patients on antimicrobials at any given time in a ward.

The study has several strengths. First, the repeated PPSs in the eight NICUs increased the robustness of our estimates of antimicrobial prescription among NICU babies. Second, we were able to capture antimicrobial prescribing practices in different types of hospitals as the eight participating hospitals represented diverse settings that are commonly seen in India. Four hospitals were stand-alone children hospitals in urban areas, two were part of a rural general hospital, and two were part of large tertiary care referral centers. Third, we examined the variation of antimicrobial use at different times of the year. Interestingly, the percentages of children on antimicrobials were not significantly different among PPSs and, thus, we did not observe any temporal variation in antimicrobial prescribing. However, the study also has several limitations. First, appropriateness of antimicrobial prescribing was not assessed, as we did not collect data on the duration of therapy, or the microbiology and antimicrobial susceptibility results. Second, although we included hospitals with different characteristics, we did not include hospitals from the public sector nor from all regions of India. Thus, our results may not be generalizable to all healthcare settings and geographic regions of the country. However, private sector accounts for more than 60% of healthcare services in India (Hauge et al., 2017). Third, as the date of the survey was chosen as per the convenience of the site principal investigator within the specified months and physicians knew that their antimicrobial prescriptions were being studied, the results might have been affected by a Hawthorne effect.

In conclusion, our study reports that half of NICU babies were on at least one antimicrobial agent and more than 70% of treatment was empiric. The recommended first-line antimicrobial agents, ampicillin and gentamicin were rarely prescribed in Indian NICUs for community acquired neonatal sepsis and pneumonia. Amikacin and other broad-spectrum agents like piperacillin-tazobactam and third generation cephalosporins were commonly used as empiric choices for community-acquired neonatal sepsis and pneumonia. Although, high resistance rates reported to first-line agents may support the use of second-line broad-spectrum agents, there is evidence that implementation of facility based treatment guidelines by incorporating diagnostic microbiological information could reduce the amount of broad-spectrum antimicrobials use and increase the use of first-line agents in Indian NICUs.
