# Supplementary material for: Point prevalence surveys of antimicrobial use among eight neonatal intensive care units in India: 2016
Source: Int J Infect Dis. 2018 Jun;71:20–4. doi: 10.1016/j.ijid.2018.03.017 (PMC5985371; doi:10.1016/j.ijid.2018.03.017)
Supplement: Supplementary file 1 [file mmc1.docx]

**Supplementary Table 1:**  **Indications for antimicrobial prescription among neonatal intensive care unit patients in India (%)**

| **Indication** | **Overall** | **Hospital A** | **Hospital B** | **Hospital C** | **Hospital D** | **Hospital E** | **Hospital F** | **Hospital G** | **Hospital H** |
| --- | --- | --- | --- | --- | --- | --- | --- | --- | --- |
| Sepsis | 49.0 | 39.0 | 73.2 | 44.4 | 38.6 | 60.8 | 35.2 | 58.1 | 70 |
| Newborn Sepsis Prophylaxis for Newborn Risk Factors | 15.6 | 18.3 | 12.2 | 0 | 0 | 3.9 | 57.4 | 2.3 | 0 |
| Proven or probable Bacterial LRTI | 12.1 | 9.8 | 0 | 22.2 | 40.4 | 0 | 5.6 | 14.0 | 0 |
| Newborn Sepsis Prophylaxis for Maternal Risk Factors | 10.1 | 4.9 | 9.8 | 0 | 3.5 | 31.4 | 0 | 20.9 | 0 |
| Treatment for Surgical disease | 4.3 | 12.2 | 4.9 | 33.3 | 0 | 0 | 0 | 0 | 0 |
| Prophylaxis for Surgical disease | 2.6 | 6.1 | 0 | 0 | 3.5 | 0 | 0 | 4.7 | 0 |
| Central Nervous System infection | 1.7 | 0 | 0 | 0 | 5.3 | 0 | 0 | 0 | 30 |
| Prophylaxis for Medical problems | 1.7 | 7.3 | 0 | 0 | 0 | 0 | 0 | 0 | 0 |
| Gastro-intestinal tract infections | 1.4 | 0 | 0 | 0 | 3.5 | 3.9 | 1.9 | 0 | 0 |
| Probable or Proven CRBI | 0.6 | 2.4 | 0 | 0 | 0 | 0 | 0 | 0 | 0 |
| Urinary Tract Infections | 0.6 | 0 | 0 | 0 | 3.5 | 0 | 0 | 0 | 0 |
| Proven or probable Viral LRTI | 0.3 | 0 | 0 | 0 | 1.8 | 0 | 0 | 0 | 0 |

CRBI, catheter-related bloodstream infection; LRTI, lower respiratory tract infection

**Supplementary Table 2: Empiric therapy prescribed for community-acquired sepsis**

| **Antimicrobial regimen** | **Number of patients (n=40)** | **% of patients** |
| --- | --- | --- |
| Piperacillin-tazobactam | 7 | 17.5 |
| Amikacin | 5 | 12.5 |
| Amikacin-Ciprofloxacin | 5 | 12.5 |
| Amikacin-Piperacillin-tazobactam | 5 | 12.5 |
| Amikacin-Cefoperazone Sulbactam | 3 | 7.5 |
| Ampicillin-Gentamicin | 2 | 5 |
| Cefoperazone Sulbactam-Gentamicin | 2 | 5 |
| Cefotaxime | 2 | 5 |
| Amikacin-Ampicillin | 1 | 2.5 |
| Amikacin-Cefotaxime | 1 | 2.5 |
| Amikacin-Ceftriaxone | 1 | 2.5 |
| Cefoperazone Sulbactam-Levofloxacin | 1 | 2.5 |
| Gentamicin | 1 | 2.5 |
| Gentamicin-Meropenem | 1 | 2.5 |
| Levofloxacin | 1 | 2.5 |
| Meropenem | 1 | 2.5 |
| Meropenem-Vancomycin | 1 | 2.5 |

**Supplementary Table 3: Empiric therapy prescribed for hospital-acquired sepsis**

| **Antimicrobial regimen** | **Number of patients (n=28)** | **% of patients** |
| --- | --- | --- |
| Piperacillin-tazobactam | 5 | 17.9 |
| Fluconazole | 3 | 10.7 |
| Amikacin-Meropenem | 2 | 7.1 |
| Amikacin-Piperacillin-tazobactam | 2 | 7.1 |
| Ceftriaxone/Cefotaxime | 3 | 7.1 |
| Aciclovir-Amikacin-Meropenem | 1 | 3.6 |
| Amikacin | 1 | 3.6 |
| Amikacin-Fluconazole-Meropenem | 1 | 3.6 |
| Ampicillin-Fluconazole-Meropenem | 1 | 3.6 |
| Ampicillin-Gentamicin | 1 | 3.6 |
| Ampicillin-Meropenem | 1 | 3.6 |
| Ciprofloxacin-Colistin | 1 | 3.6 |
| Colistin-Levofloxacin | 1 | 3.6 |
| Colistin-Meropenem | 1 | 3.6 |
| Fluconazole-Piperacillin-tazobactam | 1 | 3.6 |
| Meropenem | 1 | 3.6 |
| Meropenem-Ofloxacin | 1 | 3.6 |
| Vancomycin | 1 | 3.6 |

**Supplementary Table 4: Empiric therapy prescribed for community-acquired pneumonia**

| **Antimicrobial regimen** | **Number of patients (n=20)** | **% of patients** |
| --- | --- | --- |
| Cefotaxime | 4 | 20 |
| Piperacillin-tazobactam | 3 | 15 |
| Amikacin | 1 | 5 |
| Amikacin-Cefotaxime | 1 | 5 |
| Amikacin-Ceftriaxone | 1 | 5 |
| Amikacin-Piperacillin-tazobactam | 1 | 5 |
| Amoxicillin and enzyme inhibitor | 1 | 5 |
| Amphotericin B | 1 | 5 |
| Ampicillin-Cloxacillin- Piperacillin-tazobactam | 1 | 5 |
| Azithromycin-Ceftriaxone | 1 | 5 |
| Azithromycin-Meropenem | 1 | 5 |
| Cefotaxime-Cloxacillin | 1 | 5 |
| Colistin-Meropenem | 1 | 5 |
| Fluconazole-Linezolid- Piperacillin-tazobactam | 1 | 5 |
| Meropenem | 1 | 5 |

**Supplementary Table 5: Empiric therapy prescribed for newborn prophylaxis for newborn risk factors**

| **Antimicrobial regimen** | **Number of patients (n=33)** | **% of patients** |
| --- | --- | --- |
| Ampicillin-Gentamicin | 13 | 39.4 |
| Piperacillin-tazobactam | 3 | 9.1 |
| Amikacin | 2 | 6.1 |
| Amikacin- Piperacillin-tazobactam | 1 | 3.0 |
| Amikacin-Ciprofloxacin | 1 | 3.0 |
| Fluconazole | 1 | 3.0 |
| Ciprofloxacin | 1 | 3.0 |
| Ceftriaxone | 1 | 3.0 |
| Amikacin-Cefotaxime | 1 | 3.0 |
| Ceftazidime | 1 | 3.0 |
| Ceftriaxone-Fluconazole | 2 | 6.1 |
| Amikacin-Cefoperazone | 1 | 3.0 |
| Amikacin- Piperacillin-tazobactam | 1 | 3.0 |
| Ampicillin | 1 | 3.0 |
| Cefoperazone-Fluconazole | 1 | 3.0 |
| Cefoperazone-sulbactam | 1 | 3.0 |
| Ampicillin combinations | 1 | 3.0 |

**Supplementary Table 6: Empiric therapy prescribed for newborn prophylaxis for maternal risk factors**

| **Antimicrobial regimen** | **Number of patients (n=21)** | **% of patients** |
| --- | --- | --- |
| Ampicillin-Gentamicin | 8 | 38.1 |
| Amikacin | 5 | 23.8 |
| Amikacin- Piperacillin-tazobactam | 2 | 9.5 |
| Levofloxacin | 1 | 4.8 |
| Gentamicin | 2 | 9.5 |
| Benzylpenicillin-Gentamicin | 2 | 9.5 |
| Amikacin-Ampicillin-Metronidazole | 1 | 4.8 |
